# Interplay between Hepatitis C Virus and Redox Cell Signaling

**DOI:** 10.3390/ijms14034705

**Published:** 2013-02-26

**Authors:** Anna Ruggieri, Simona Anticoli, Lucia Nencioni, Rossella Sgarbanti, Enrico Garaci, Anna Teresa Palamara

**Affiliations:** 1 Department of Infectious, Parasitic and Immune Mediated Diseases, Italian National Institute of Health, 00161 Rome, Italy; 2 Department of Public Health and Infectious Diseases, Institute Pasteur, Cenci-Bolognetti Foundation, “Sapienza” University of Rome, 00185 Rome, Italy; E-Mails: simona.anticoli@gmail.com (S.A.); lucia.nencioni@uniroma1.it (L.N.); annateresa.palamara@uniroma1.it (A.T.P.); 3 San Raffaele Pisana Scientific Institute for Research, Hospitalization and Health Care, 00163 Rome, Italy; E-Mails: rossella.sgarbanti@uniroma1.it (R.S.); garaci@uniroma2.it (E.G.)

**Keywords:** HCV, JFH-1, antioxidants, oxidative stress, GSH, MAPK, Akt

## Abstract

Hepatitis C virus (HCV) infects approximately 3% of the world’s population. Currently licensed treatment of HCV chronic infection with pegylated-interferon-α and ribavirin, is not fully effective against all HCV genotypes and is associated to severe side effects. Thus, development of novel therapeutics and identification of new targets for treatment of HCV infection is necessary. Current opinion is orienting to target antiviral drug discovery to the host cell pathways on which the virus relies, instead of against viral structures. Many intracellular signaling pathways manipulated by HCV for its own replication are finely regulated by the oxido-reductive (redox) state of the host cell. At the same time, HCV induces oxidative stress that has been found to affect both virus replication as well as progression and severity of HCV infection. A dual role, positive or negative, for the host cell oxidized conditions on HCV replication has been reported so far. This review examines current information about the effect of oxidative stress on HCV life cycle and the main redox-regulated intracellular pathways activated during HCV infection and involved in its replication.

## 1. Introduction

According to evaluations of the World Health Organization (WHO), hepatitis C virus (HCV) currently infects at least 130 million people worldwide, which is about 3% of the global population [1]. Following acute HCV infection, a chronic state is established in as many as 80% of infected individuals. Although many subjects carrying the virus remain asymptomatic, chronicity is often accompanied by altered liver function and progressive liver disease that culminates in cirrhosis and hepatocellular carcinoma in up to 20% of chronically infected individuals [2].

HCV is a positive-sense single stranded RNA virus of the *Flaviviridae* family. The HCV genome is about 9.6 kb in length and consists of the 5′ untranslated region (UTR), the structural (core, E1 and E2) and non-structural (p7, NS2, NS3, NS4 A/B, NS5 A/B) proteins and the 3′ UTR. HCV binds to the CD81 and/or SR-BI receptors, enters the cell through clathrin-mediated endocytosis and is uncoated, releasing the RNA genome into the cytoplasm. The positive-sense RNA first serves as a messenger RNA for translation of a single large polyprotein that is proteolytically cleaved, by host and viral peptidases, to generate individual proteins. Subsequently, HCV RNA acts as a template for viral genome replication, mediated by virus encoded RNA-dependent RNA polymerase, NS5B, in cooperation with NS5A and other viral proteins. The virus replication complex is localized to the membranous web that is formed by modified membranes of the ER and Golgi. After encapsidation of the viral genome, virions are exported through the host secretory pathway. For recent reviews on the HCV life cycle see [3,4].

Current therapy of chronic hepatitis C is based on a combination of PEG-interferon (PEG-IFN)-α and ribavirin. This combination therapy is not fully effective, as it achieves a sustained virological response (SVR) in less than half of treated patients with HCV genotype 1 and about 80% of those with genotype 2, and causes severe side effects [5–7]. Recent approval, in Europe and in the United States, of the two new drugs targeting the HCV protease (telaprevir and boceprevir) produced an increased SVR rate to 70%–80% in triple combination therapy with PEG-IFN-α and ribavirin [8]. However triple combination treatment has limitations in partial non-responders and null responders to a prior course of PEG-IFN-α and ribavirin [9–12] and has more severe side effects.

From the above, a pressing need exists for the development of new and alternative therapeutic strategies to combat HCV infection. To circumvent the onset of drug resistance, a major obstacle to therapeutic success, it seems to be particularly important to approach viral infection therapy by considering the interactions between virus and host cells. In fact, current opinion is to target antiviral drug discovery to the host cell pathways on which the virus relies, instead of against viral structures, in order to decrease the likelihood of acquiring drug resistance [13,14].

It is known that many cellular proteins involved in a wide range of signaling pathways that are manipulated by HCV, to promote its own replication [15–18], are influenced by the intracellular redox state [19–21]. The redox state of a cell results from an equilibrium between the production of reactive oxygen/nitrogen species (ROS/RNS) and the anti-oxidative defenses (antioxidant molecules and enzymes) [21]. An imbalance towards pro-oxidative conditions is referred to as oxidative stress. The intracellular thiols are an important class of antioxidant molecules, with glutathione (GSH) being the most abundant. In cells, GSH can be free or bound to proteins. Free GSH exists mainly in its reduced form (GSH), which can be transformed to the oxidized gluthatione (GSSG) during oxidative stress and it can be reverted to the reduced form by the enzyme GSH reductase. The ratio between GSH/GSSG is considered an index of the antioxidant capacity of the cell [22]. The reduced form of glutathione is an important radical scavenger that directly neutralizes a variety of reactive molecules (such as superoxide anion and hydroxyl radicals) [23]. In addition to its role as a redox buffer, GSH can form mixed disulfides (GSSR) with protein thiols (*S*-glutathionylation), thus protecting cysteine residues of proteins from irreversible oxidation events. Moreover, *S*-glutathionylation may alter protein function and is actually considered to be involved in signal transduction [24,25]. It has been demonstrated that infections by several viruses, including HCV, are frequently characterized by host-cell redox changes, characteristic of oxidative stress, that affect the efficacy of viral replication [26–33].

This review presents a summary of the current knowledge about redox regulation of HCV life cycle and discusses some of main redox regulated intracellular pathways activated during HCV infection and involved in its replication.

## 2. HCV Infection Induces Oxidative Stress

Increased oxidative stress is a hallmark of HCV infection (reviewed in [28,29,33]). Clinical studies have shown that HCV patients have elevated levels of reactive aldehydes produced by lipid peroxidation, such as malondialdehyde and 4-hydroxy-2-nonenal, in their serum, peripheral blood mononuclear cells and liver [34,35]. Liver levels of 8-hydroxydeoxyguanosine, a marker of oxidative damage, are also elevated [34,36]. Furthermore, an increased glutathione turnover in the liver, blood and lymphatic system has been suggested by the observation of decreased ratio between GSH/GSSG [34,37–39].

**Figure 1:**
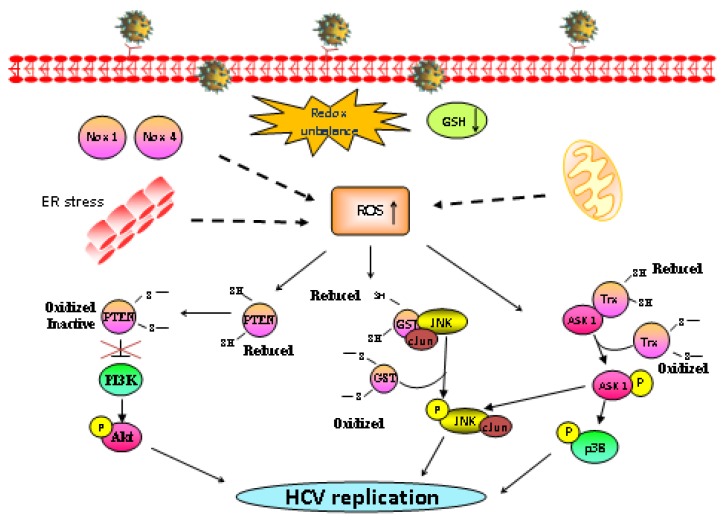
Schematic representation of ROS sources during Hepatitis C virus (HCV) infection and redox mechanisms for activation of MAPKs and PI3K/Akt.

Usually, viruses induce an inflammatory response at the site of infection, where release of ROS by immune cells acts as non-specific toxins, kills pathogens and injuries adjacent cells. In the case of persistent infections, chronic inflammation results in overwhelming production of ROS that induces tissues damage and predispose to disease induction [40]. However, although inflammation is an important source of ROS during chronic infection [41,42], HCV infected patients with minimum or no liver disease showed markers of oxidative stress [43] and HCV transgenic mice exhibited signs of increased oxidative damage in the absence of inflammation [44]. These evidences suggest that HCV may directly promote oxidative stress in hepatocytes (Figure 1). Actually, numerous *in vitro* studies have shown that not only HCV replication, but also expression of the HCV structural and non-structural proteins, directly act as inducers of oxidative stress, mainly through mitochondrial dysfunction of hepatocytes and endoplasmic reticulum (ER) stress [45–52]. In particular, direct interaction of the structural HCV core protein with mitochondria decreases the mitochondrial NADPH levels, reduces the activity of the electron transport complex I and increases generation of ROS [45–47]; the non-structural proteins NS5A [49,50], NS4B [50] and the structural glycoproteins E1 and E2 [51] induce ROS production by ER stress. Recently, it has been demonstrated that hepatocyte NAD(P)H oxidase (Nox) proteins 1 and 4 represent another prominent source of ROS during HCV infection [53,54], as they were found significantly elevated both in HCV infected cells and in the liver of HCV-infected patients [53]. In addition, HCV replication may induce impairment of host cell antioxidant defenses [55–58], and directly alter the endogenous levels of GSH [53,56,59] (Table 1). Although *in vivo* studies reported a decreased hepatic GSH content in HCV chronically infected patients [34,38,40], however studies *in vitro* showed conflicting results about the effect of HCV on intracellular GSH as well as on the enzymes regulating GSH homeostasis [55,60–62]. In this regard, Roe and collaborators [59] reported a significant raise of GSSG in HCV infected cells; conversely, increased GSH concentration was demonstrated by de Mochel *et al*. [53] using same *in vitro* infection system. Analogous contradiction has been described for the effect of HCV on the activity of NF-E2-related factor 2 (Nrf2), a central regulator of the enzymes of glutathione homeostasis [63,64]. Recently, two studies, performed by Burdette *et al*. [60] and Ivanov *et al*. [61] reported activation of Nrf2 pathway, respectively in HCV infected Huh-7 cells and in cells expressing the individual viral proteins. Conversely, Carvajal-Yepes and collaborators [62] demonstrated suppression of this pathway upon transfection of cells with HCV clones. The apparently disparate effects on GSH by HCV may be due to different experimental systems used (HCV transfected *versus* infected cell cultures). However, it has to be considered that viruses producing chronic infections, such as HIV and HCV, cause significant changes of GSH levels only after chronic infection is well established [27,65]. So, it has been proposed [61] that data by Burdette *et al*. [60] and by Ivanov *et al*. [61] may be representative of the acute phase of HCV infection, when the cells enhance the expression of antioxidant genes to protect themselves against the virus induced oxidative stress. In contrast, data from the study of Carvajal-Yepes [62] could be related to chronic virus infection. Further studies about the changes of GSH levels during HCV acute and chronic infections are required to clarify this point.

## 3. Oxidative Stress Affects HCV Replication

HCV related oxidative stress is involved in the development of liver injury, and in the progression of liver disease towards fibrosis, cirrhosis and HCC [28,29,66,67]. Therefore, antioxidants have been proposed as new potential treatment of HCV patients. For example, in a phase I clinical trial testing a combination of seven different antioxidants, 44% of the patients had normalization of liver enzymes levels and 36.1% of the treated patients had histological improvement [68]. Alongside this positive effect of the anti-oxidants, however, it remains unclear how modulation of oxidative stress can affect HCV replication. In general, treatment with drugs that amplify the effects of endogenous ROS, generated during normal cellular metabolism or in response to HCV, has suppressive effect on HCV replication. Using replicon cells (that are human hepatoma cell lines constitutively replicating HCV RNA but not producing infectious virus particles), Choi and collaborators found that H_2_O_2_ and t-butylhydroperoxide (t-BOOH), which generates low levels of intracellular ROS, reduced HCV RNA by a mechanism involving the disruption of HCV replication complex on cell membranes [69,70]. Similar suppression of intracellular HCV was obtained upon ROS induction with glucose oxidase (GO) transfection in cell lines [71]. In addition, modulation of intracellular glutathione, by arsenic trioxide or by BSO, negatively affected HCV replication *in vitro*, either in replicon cells as well as in cells infected with HCV infectious clone (JFH1-infected cells) [71,72]. According to the above-reported negative effect of oxidative stress on HCV *in vitro*, some anti-oxidants (such as vitamins A, vitamin E and resveratrol) have been shown to enhance HCV replication in replicon model, with yet unknown mechanisms [73,74]. On the other side, opposite effect of some antioxidant treatments on HCV has also been reported. For examples, *N*-acetylcysteine (NAC) [75], pyrrolidine dithiocarbamate (PDTC) [76,77], sylimarin [78,79], naringerin [80–82], quercetin [82,83], curcumin [84] and also the acetylsalicylic acid (ASA) [77], have been found to decrease HCV replication and HCV proteins level *in vitro*. In addition, transient transfection of the anti-oxidant enzyme, Mn-superoxide dismutase, in replicon cells lowered viral replication [76]. The steps in virus life cycle targeted by these antioxidants and the mechanisms related to their action are mostly unknown, although in some cases they have been described. As examples, PDTC is reported to act through inhibition of MAPK pathways [76]; sylimarin inhibits virus entry and infectious virions production into culture supernatants through the inhibition of microsomal triglyceride transfer protein (MTP) activity and apolipoprotein B secretion [78]; naringerin blocks virus assembly, partly by the activation of PPARα, and consequent decrease in VLDL production [80]. Few clinical studies describing the effect of antioxidant supplementation on HCV titer are available and have yielded so far inconsistent results [68,85,86]. This lack of a clear correlation between oxidative stress and HCV replication remains an open question and may in part be explained by the fact that the same antioxidant molecules can display pro- or anti-oxidant functions, depending on their own oxidative status, which in turn reflects the specific redox potential of the microenvironment [87,88]. In support of these considerations, Wang and collaborators found that pre-treatment of mice with NAC, a GSH precursor, prevents liver from acute ethanol-induced damage, via counteracting ethanol-induced oxidative stress; whereas, when administered after ethanol, NAC behaves as a pro-oxidant and exacerbates acute ethanol-induced liver damage [89]. Furthermore, we have shown that resveratrol (RV) can decrease GSH in a different virus and cell system, thus functioning as oxidant molecule [90]. This fact has been explained considering that, although RV can quench reactive free radicals by donating hydrogen atoms, this process generates also phenoxyl radicals that can oxidize GSH to GS·. Moreover, oxidation of the RV-phenoxyl radical produces an RV-quinone form, which can alkylate GSH, further diminishing the intracellular concentrations of free GSH [91]. A similar behaviour has been shown by vitamin E (tocopherol), a chain-breaking antioxidants that interferes with radical chain propagation by trapping radicals. During its metabolism, vitamin E is converted into tocopheroxyl radical with low reactivity that needs to be removed by a secondary antioxidant, such as ascorbic acid (vitamin C). If the removal of tocopheroxyl radical by a secondary antioxidant is delayed, it may promote lipid peroxidation, functioning as pro-oxidant [92].

Therefore antioxidants are considered intriguing molecules to be used as antivirals. However most of the studies so far available, evaluate the antiviral potential of antioxidant molecules, without concurrent measure of their effect on the cellular redox state, which could be the reason behind reported controversies in literature.

## 4. Redox-Regulated Signaling Pathways: MAPK and PI3K/Akt Signaling Pathways Are Critical Controllers of HCV Replication

An abundance of scientific literature exists demonstrating that oxidative stress influences several signaling pathways [25,93–95], among which the two mostly affected, MAPK (Mitogen Activated Protein Kinase) and PI3K/Akt pathways, have a pivotal role on replication of several viruses, such as influenza A virus [96–98], HIV [99], human cytomegalovirus [100], varicella-zoster [101] and also HCV [15,17]. MAP kinases (comprising the three best characterized members ERK, JNK and p38 MAPK) and Akt are activated by general induction of intracellular ROS [93,94,102] and are inhibited by the antioxidants [17,76,103]. Moreover, exposure of cells to exogenous H_2_O_2_, that mimic oxidative stress, also leads to activation of MAPK and PI3K/Akt pathways in multiple cell types (vascular smooth muscle cell, cardiac myocytes, A431 cell, CHO cell line, hepatocytes) [94,104–109]. Different mechanisms have been proposed for activation of the two pathways in response to ROS [19,25,93–95] (Figure 1). In normal redox conditions, thioredoxin (TRX) association with ASK1, the MAKKK for JNK and p38, maintains the pathways inactive [110,111]; upon oxidative stress thiol modification of TRX, promotes its dissociation from ASK1 [93,94] thus activating p38 and JNK [112]. A second proposed mechanism involves ROS induced oligomerization of glutathione-*S*-transferase Pi (GSTp), resulting in its dissociation from the complex with JNK and the subsequent activation of the pathway [113]. A further mechanism for activation of MAPK and Akt is by degrading the protein tyrosine phosphatases, PtPases, that maintain the pathways in inactive state [19,25]. An example of this latter is oxidation and consequent inactivation of PTEN, a negative regulator of Akt that results in PI3K/Akt activation [114,115].

It is known that during its life cycle, HCV activates MAPK and Akt pathways that have a role in the pathogenesis of inflammation, fibrosis, HCC [116–120] and in viral immune evasion strategies occurring during HCV infection [17,121]. With regard to the role of MAPK cascades in HCV replication, virus binding to the CD81 receptor has been shown to activate the Raf/MEK/ERK pathway that was necessary for post-entry events [122]. Consistently, it has recently been proposed that the blockade of ERK cascade with the inhibitor of MEK 1/2 (U0126) prevents HCV assembly and virion release [16,123]. Furthermore, the inhibitors of JNK and p38MAPK (SP600125 and SB203580 respectively) blocked HCV replication in replicon systems, suggesting that these kinases are tightly involved in positive control of HCV life cycle [76]. However, some authors suggested a negative role of ERK1/2 pathway on HCV *in vitro*, as activation of this pathway was shown to suppress viral replication in replicon system, whereas its inhibition promoted HCV replication [124–126]. The discrepancies on the effects of the diverse cascades of the MAPK pathway (ERK, JNK and p38) on HCV replication, are probably due to the different *in vitro* system used by the authors, therefore further research would need to elucidate this issue.

With regard to the role of PI3K/Akt pathway several reports have shown that it is activated by HCV [15,119,120] and that this event is critical for viral replication, as suggested by studies with small interfering RNA (siRNA) [127] or compounds that specifically block Akt activity [18,84,127]. Among these latter, triciribine, that blocks Akt activity without affecting its upstream activators, was found to inhibit both basal HCV replication, as well as that enhanced by epithelial growth factor (EGF) [127]. Conversely, little is known about the steps of HCV replication cycle influenced by PI3K/Akt. Recently, Liu *et al.*[18] demonstrated that HCV rapidly and transiently activated Akt, to enhance its entry into the cells, via the interaction between HCV E2 envelope protein and its co-receptors, CD81 and claudin-1. Interestingly, PI3K/Akt pathway positively affects HCV replication by modulating lipid metabolism; the mechanisms involve enhanced expression of SREBP-1 (sterol regulatory element binding protein-1) [84], an important transcription factor of lipogenic gene expression, and inhibition of the AMPK (AMP-activated protein kinase) activity [128], that results in stimulation of cholesterol and triglyceride synthesis. Increasing evidences show that HCV is critically dependent on cellular lipids throughout its life cycle [4,129,130]. In fact viral RNA replication complexes localize to lipid rafts (membranous structures derived from the ER) rich in sphingomyelin and cholesterol [131]; moreover a crucial role for lipid droplets (LDs) in HCV assembly has also been demonstrated [132].

On the basis of all the above reported data it could be reasonably speculated that PI3K/Akt pathway, through modulation of hepatic lipid metabolism, could have a role both in early and late stages of HCV life cycle. However, further investigations would need to disclose the exact steps of HCV life cycle influenced by Akt pathway.

## 5. Conclusions

Although HCV induces oxidative stress in host cells, all current revised literature points to a dual role of the host cell oxidized conditions on HCV replication. Several studies are in favor of a positive control of HCV replication by oxidative stress; however, it cannot be ruled out that virus induced oxidative stress can negatively modulate its own replication. This could be a mechanism to control the interaction at equilibrium between virus and host cell, which is the basis for establishment of chronicity. Although much has yet to be disclosed, the redox-mediated host cell mechanisms so far identified, together with those that will be further recognized, could uncover new effective approaches for HCV treatment.

## Figures and Tables

**Table 1:** Mechanisms of oxidative stress induction by HCV infection and viral proteins involved.

Source of ROS/RNS	HCV proteins	References
Transcriptional up-regulation of iNOS	core, NS3	[47]
Activation of Nox2 in PBMCs	NS3	[41]
GSH depletion	Core	[56]
Increased production of mitochondrial ROS by the electron transport chain	Core	[45]
Activation of Nox2 in Kupffer cells	HCV	[29,40]
Endoplasmic reticulum stress (ER stress)	NS4B, NS5A, E1, E2	[49–51]
Increase of proinflammatory cytokines	HCV, core	[54]
Activation of Nox1 and 4 proteins in hepatocytes	HCV	[53,54]
